# Automated Bergmann-Kliesch Score assessment in testicular biopsies using artificial intelligence-based whole-slide image analysis: a retrospective monocentric development and validation study

**DOI:** 10.1186/s12610-026-00329-x

**Published:** 2026-10-06

**Authors:** Simon Gassen, Nadine Flinner, Ingvild Frøberg Mathisen, Julia Bein, Ole Beldermann, Lukas Haug, Jens Köllermann, Felix Chun, Annette Bachmann, Kerstin Lehr, Robin S. Mayer, Peter J. Wild

**Affiliations:** 1 Dr. Senckenberg Institutes of Pathology, Neuropathology and Human Genetics, Goethe University Frankfurt, Frankfurt am Main, Germany; 2 Department of Urology, University Medical Center Frankfurt, Goethe University Frankfurt, Frankfurt am Main, Germany; 3 Department of Obstetrics and Gynecology, University Medical Center Frankfurt, Goethe University Frankfurt, Frankfurt am Main, Germany

**Keywords:** Bergmann-Kliesch Score, Testicular biopsy, Spermatogenesis, Non-obstructive azoospermia, TESE, ICSI, Deep learning, Digital pathology, Whole-slide image analysis, Image registration, Score de Bergmann-Kliesch, Biopsie testiculaire, Spermatogenèse, Azoospermie non obstructive, TESE, ICSI, Apprentissage profond, Pathologie numérique, Analyse d’images histologiques entières, Recalage d’images

## Abstract

**Background:**

The Bergmann-Kliesch Score is a histopathological grading system used to evaluate spermatogenesis in testicular biopsies, quantifying the fraction of seminiferous tubules containing elongated spermatids. It serves as a key parameter in the clinical process of men with non-obstructive azoospermia (NOA) undergoing testicular sperm extraction (TESE) for subsequent intracytoplasmic sperm injection (ICSI). Manual Bergmann-Kliesch Score assessment is time-consuming and subject to inter- and intraobserver variability. This study aimed to develop and validate an artificial intelligence (AI)-based computational pipeline for automated Bergmann-Kliesch Score assessment from digitized testicular biopsy whole-slide images (WSIs).

**Results:**

A retrospective monocentric cohort of 74 patients who underwent TESE at the University Medical Center Frankfurt was analyzed. A dual-model machine-learning pipeline was used to process paired haematoxylin-eosin (HE) and OCT3/4 immunohistochemically stained WSIs. A U-Net architecture with a ResNet-34 backbone was trained on 60 HE-stained slides for tubular segmentation, monitored by a multi-class Dice coefficient of 0.897 on the validation set. A second U-Net model with a ResNet-34 backbone was trained on 15 OCT3/4-stained slides comprising 14,226 manually annotated elongated spermatids for spermatid detection. Both modalities were integrated via multimodal image registration combining rigid alignment and B-spline warping, enabling spatial projection of spermatid detections onto tubular segmentations. On the patient-level held-out test set of 15 patients, AI-derived Bergmann-Kliesch Scores showed strong agreement with pathologist-assigned reference scores (Pearson *r* = 0.957, Spearman ρ = 0.856, R^2^ = 0.916, mean absolute error = 0.93, root mean squared error = 1.28). At the clinical cutoff of Bergmann-Kliesch Score ≥ 1, the pipeline correctly classified all test-set cases. Across the full evaluation cohort of 74 patients, agreement remained robust (Pearson *r* = 0.851, Spearman ρ = 0.829, mean absolute error = 1.52, root mean squared error = 2.20).

**Conclusions:**

This AI-based pipeline enables automated, reproducible Bergmann-Kliesch Score assessment from routinely stained testicular biopsy WSIs, demonstrating strong agreement with manual pathologist evaluation. The system shows promise as a clinical decision-support tool in TESE workflows. External multicentric validation and prospective correlation with ICSI outcome data are warranted in future studies.

**Supplementary information:**

The online version contains supplementary material available at https://doi.org/10.1186/s12610-026-00329-x.

## Résumé

**Contexte:**

Le Score de Bergmann-Kliesch est un système de classification histopathologique utilisé pour évaluer la spermatogenèse dans les biopsies testiculaires, en quantifiant la fraction de tubules séminifères contenant des spermatides allongées. Il constitue un paramètre essentiel dans la prise en charge clinique des hommes atteints d’azoospermie non obstructive (ANO) qui bénéficient d’une extraction testiculaire de spermatozoïdes (TESE) en vue d’une injection intracytoplasmique de spermatozoïdes (ICSI). L’évaluation manuelle du Score de Bergmann-Kliesch est chronophage et soumise à une variabilité inter- et intra-observateur. Cette étude visait à développer et à valider un pipeline computationnel basé sur l’intelligence artificielle (IA) pour le calcul automatisé du Score de Bergmann-Kliesch à partir d’images de lames histologiques entières (WSI) numérisées de biopsies testiculaires.

**Résultats:**

Une cohorte rétrospective monocentrique de 74 patients ayant bénéficié d’une TESE au Centre Hospitalier Universitaire de Francfort-sur-le-Main a été analysée. Un pipeline d’apprentissage automatique à deux modèles a été utilisé pour traiter des WSI appariées colorées à l’hématoxyline-éosine (HE) et marquées par immunohistochimie OCT3/4. Une architecture U-Net avec un backbone ResNet-34 a été entraînée sur 60 lames colorées à l’HE pour la segmentation tubulaire, atteignant un coefficient de Dice multi-classes de 0,897 sur l’ensemble de validation. Un second modèle U-Net avec un backbone ResNet-34 a été entraîné sur 15 lames marquées à l’OCT3/4 comprenant 14 226 spermatides allongées annotées manuellement pour la détection des spermatides. Les deux modalités ont été intégrées par recalage d’images multimodal combinant un alignement rigide et une déformation par B-splines, permettant la projection spatiale des détections de spermatides sur les segmentations tubulaires. Sur l’ensemble de test de validation au niveau patient comprenant 15 patients, les Scores de Bergmann-Kliesch dérivés de l’IA ont montré une forte concordance avec les scores de référence assignés par les pathologistes (coefficient de Pearson *r* = 0,957; coefficient de Spearman ρ = 0,856; R^2^ = 0,916; erreur absolue moyenne = 0,93; erreur quadratique moyenne = 1,28). Au seuil clinique de Score de Bergmann-Kliesch ≥ 1, le pipeline a correctement classé tous les cas de l’ensemble de test. Sur l’ensemble de la cohorte, la concordance est restée robuste (coefficient de Pearson *r* = 0,851; coefficient de Spearman ρ = 0,829; erreur absolue moyenne = 1,52; erreur quadratique moyenne = 2,20).

**Conclusions:**

Ce pipeline basé sur l’IA permet l’évaluation automatisée et reproductible du Score de Bergmann-Kliesch à partir de WSI de biopsies testiculaires colorées en routine, démontrant une forte concordance avec l’évaluation manuelle par les pathologistes. Le système se révèle prometteur en tant qu’outil d’aide à la décision clinique dans les workflows de TESE. Une validation externe multicentrique ainsi qu’une corrélation prospective avec les données de résultats des ICSI sont justifiées dans de futures études.

## Introduction

Male infertility affects an estimated 30 million men worldwide [1]. Males are considered solely responsible for 20–30% of infertility cases with their contribution to all cases being an estimated 50% [1]. Non-obstructive azoospermia (NOA) is defined as the absence of spermatozoa in the ejaculate due to impaired spermatogenesis rather than ductal obstruction [2]. NOA is viewed as one of the most severe manifestations of male infertility [3]. In men with NOA, testicular sperm extraction (TESE) combined with intracytoplasmic sperm injection (ICSI) represents the only established treatment option for achieving biological parenthood [4].

However, sperm retrieval rates following TESE are limited, with a recent meta-analysis of 117 studies reporting an overall success rate of approximately 47% (95% CI: 45–49%) [5]. TESE is an invasive procedure that carries the risk of testicular damage, hormonal sequelae, and postoperative complications [6]. Tissue obtained through TESE is partially cryopreserved for potential later use and partially sent to pathology for microscopic analysis. Accurate assessment of testicular histology is therefore of considerable clinical importance, as it determines the likelihood that the cryopreserved tissue contains viable spermatozoa and thereby informs the decision to proceed to ICSI. An incorrect score risks exposing the female partner to an unnecessary, invasive procedure without a realistic chance of success. Among several methods to analyze the histopathology of testicular biopsies in men with NOA, we focus on the Bergmann-Kliesch Score, an established quantitative grading system [7]. The score quantifies the fraction of seminiferous tubules containing one or more elongated spermatids among all evaluated seminiferous tubules in a whole-slide. The result is determined as the percentage of tubules with elongated spermatids, given as an absolute score between 0 and 10 [7]. Germ cell aplasia or Sertoli cell-only syndrome (SCOS), characterized by absent spermiogenesis, is the most common cause of NOA and is indexed by a Bergmann-Kliesch Score of 0 [8]. Furthermore, a score between 1 and 7 represents the pattern of reduced spermiogenesis to a varying degree. This pattern is described as mixed atrophy indicating that the testis retains partial spermatogenic capacity [9]. The classification of regular spermiogenesis dictates 80–100% (score between 8 and 10) of evaluated seminiferous tubules in a whole-slide containing elongated spermatids according to Bergmann and Kliesch [7]. A subclassification of predominant atrophy of the germinal epithelium with isolated formation of elongated spermatids is represented with decimal scores of 0.1 to 0.9 [7]. The outcome of quantitatively grading the histopathological testicular biopsies informs the clinical recommendation for or against the execution of ICSI. The recommendation for ICSI is made if the score is at or above the clinical decision cutoff of ≥1.

However, manual Bergmann-Kliesch Score determination is presumed to be susceptible to substantial inter- and intraobserver variability. This is based on the evidential demonstration that the coarse qualitative interpretation of testicular histopathology among pathologists shows only moderate interobserver agreement (κ = 0.43) [10]. In addition, the infrequent exposure and lack of homogeneous reporting terminology further compound this variability [11]. Peyrin-Biroulet et al. and Balkenhol et al. showed that the quantitative assessment of histological images from the gastrointestinal system and the breast, respectively, is equally prone to inter- and intraobserver variability, suggesting that this is a fundamental limitation of manual histopathological scoring across organ systems [12, 13]. Given the clinical importance of the Bergmann-Kliesch Score in determining TESE decisions in men with NOA, and its susceptibility to the same variability, an automated and reproducible method for its assessment is warranted. In both studies, AI-based automation proved effective in standardizing quantification and improving reproducibility [12, 13].

AI in andrology is rapidly emerging, with applications in data processing, surgical management and diagnostic imaging [14, 15]. Many of the first clinical AI applications use non-histological modalities such as ultrasound-based methods to predict sperm retrieval outcomes in patients with azoospermia or automated identification of sperm cells in micro-TESE suspensions [16, 17]. Furthermore, AI-based classification approaches for histological modalities have since been explored as well. Ito et al. demonstrated AI-based classification of testicular histopathology by Johnsen Score [18]. Automated per-tubule analysis of testicular WSIs has also been shown in preclinical settings, including deep-learning-based spermatogenic staging in non-human primates [19] and cell- and tubule-level classification in rodents [20, 21]. More recently, Kockwelp et al. introduced MARTHA, a deep-learning framework combining passive eye-tracking with neural networks for quantitative analysis of human testicular histology in whole-slide images [22]. To the best of our knowledge, no fully automated, WSI-based, per-tubule pipeline for the clinical Bergmann-Kliesch Score in human diagnostic biopsies has been reported to date.

In this study, we developed and validated a fully automated computational pipeline for the assessment of the Bergmann-Kliesch Score from whole-slide images of testicular biopsies.

## Methods

### Study design and patient cohort

We conducted a retrospective study with a monocentric cohort of 84 adult male patients with non-obstructive azoospermia who underwent TESE at the University Medical Center Frankfurt between January 2019 and October 2024, as recorded in the case management system of the Senckenberg BioBank (SBB) of the Dr. Senckenberg Institutes of Pathology, Neuropathology and Human Genetics. Exclusion criteria included samples with exclusively epididymal tissue without testicular parenchyma or testicular parenchyma without seminiferous tubules, precluding evaluation of seminiferous tubules. Ten patients were excluded for these reasons. The final analysis cohort comprised 74 patients.

### TESE procedure

Tissue routinely obtained through conventional open TESE at the Department of Urology, University Medical Center Frankfurt was taken from two areas of each of the two testicles (cranial and caudal pole). Without microsurgical magnification, four samples were taken per area, totaling 16 samples. Three specimens were cryopreserved for potential future ICSI with one specimen being routed to the Department of Pathology for further histopathological examination. The Department of Pathology processed and evaluated the tissue by assessing the Bergmann-Kliesch Score for each specimen received. The score was then forwarded to the Andrology Unit of the University Medical Center Frankfurt, where the recommendation for proceeding to use the remaining cryopreserved tissue for ICSI was made upon the Bergmann-Kliesch Score being equal to or greater than one (i.e., at least 10% of seminiferous tubules containing one or more elongated spermatids).

### Datasets and tissue processing

Two datasets were utilized for training and evaluation of the two image segmentation models developed in this study. The first dataset was derived from the slides sourced from the archive of the SBB and was used exclusively for model development. Sixty haematoxylin-eosin-stained slides from 58 patients were included for the training of the tubular segmentation model. Two patients contributed two sections each from different anatomical regions. The tubular segmentation model’s purpose was solely to segment the seminiferous tubules in the evaluated area of a WSI.

Fifteen OCT3/4 immunohistochemically stained slides without positive immunoreactivity for germ cell neoplasia in situ (GCNIS) from 15 patients were included for the training of the spermatid detection model. The spermatid detection model’s purpose was solely to detect the elongated spermatids in the evaluated area of a WSI. To facilitate consistent and reliable identification of elongated spermatids during annotation, fully automatic OCT3/4 immunostaining was selected as it provides enhanced visual contrast for these small structures compared to haematoxylin-eosin (HE) alone (Additional file 1), despite OCT3/4 not being a specific marker for late spermatid stages in its conventional diagnostic application [23].

**Fig. 1:**
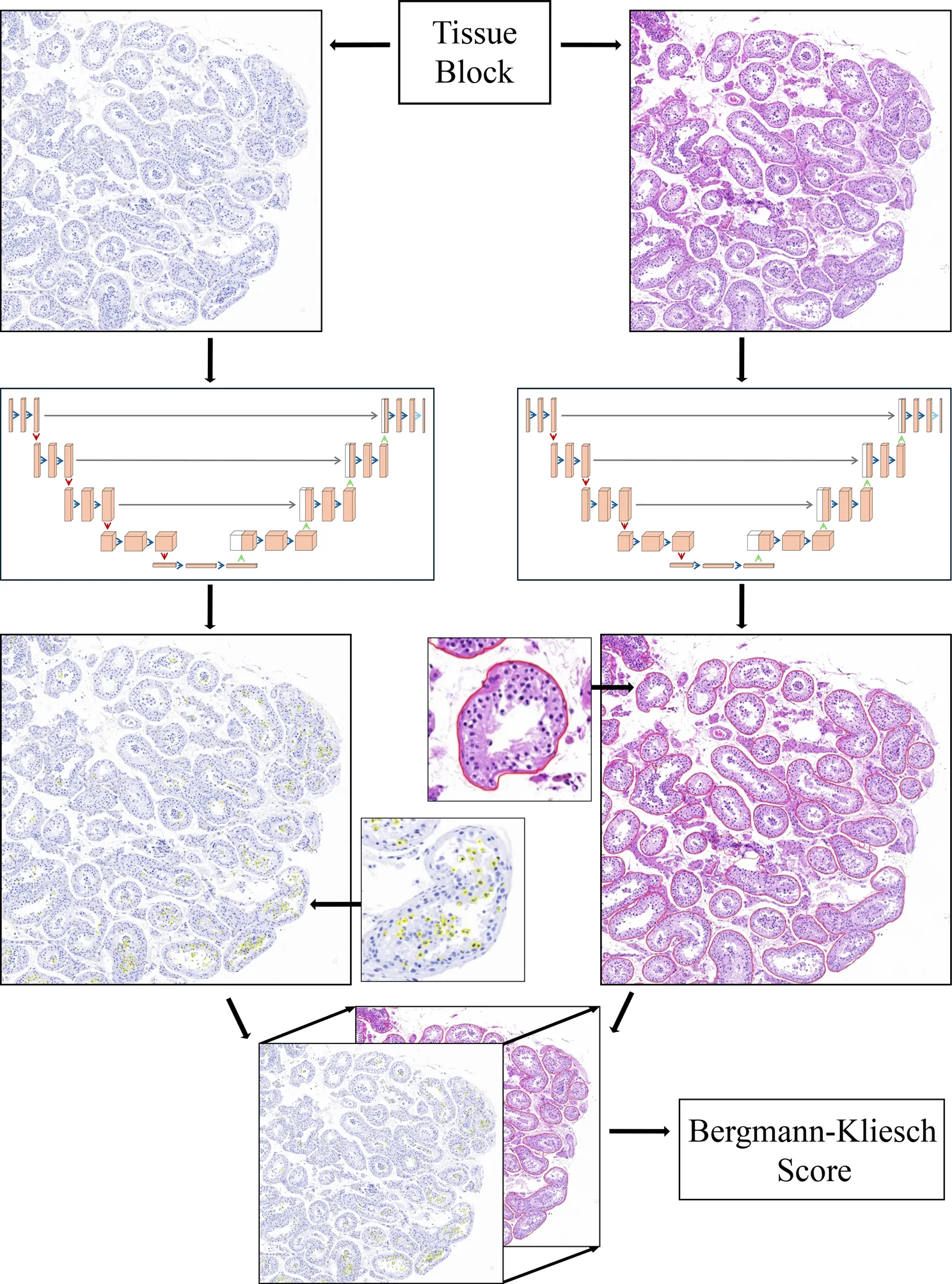
“Schematic overview of the dual AI-based pipeline for automated Bergmann-Kliesch Score assessment.” Legend: “For each tissue block, two consecutive serial sections were prepared. One cut was stained with haematoxylin-eosin (HE, right side) and one was OCT3/4 immunohistochemically stained (left side). Each modality was processed by an independent U-Net segmentation model with a ResNet-34 encoder backbone (schematic illustration). The HE model segmented seminiferous tubules. The OCT3/4 model detected elongated spermatids. Multimodal image registration projected the spermatid detections onto the tubular segmentations. The Bergmann-Kliesch Score was then computed as the proportion of tubules containing at least the adopted threshold of detected spermatids, divided by 10, yielding values between 0 and 10”

The second dataset was created exclusively for this study. One piece of formalin-fixed, paraffin-embedded (FFPE) tissue of every retrospectively included patient was chosen at random out of the SBB’s archive. For every piece of tissue, two consecutive serial section cuts were made, with the section thickness being 2 μm. The first histological cut was HE-stained, and the second histological cut was immunohistochemically stained against OCT3/4 (clone N1NK). Both stainings were performed using the same standard institutional protocol as the stained WSIs utilized for the first dataset. The slides were then scanned at a 40x magnification, yielding WSIs with a pixel resolution of 0.2486 µm/pixel at the native pyramid level (Pannoramic P1000, 3DHISTECH Ltd., Budapest, Hungary). The direct adjacency of the serial sections preserved morphological correspondence between the paired WSIs, enabling reliable image registration. The image segmentation models created with the first dataset were then used to segment the seminiferous tubules and the elongated spermatids on this second dataset. After image segmentation, the two modalities were aligned by multimodal image registration, allowing spermatid detections to be projected into coordinates corresponding to the HE stained slides. The Bergmann-Kliesch Score was then computed automatically from the spatial intersection of spermatid detections with seminiferous tubule polygons. Figure 1 shows a schematic overview of the dual AI-based pipeline.

Two evaluation sets were defined from the second dataset. The first comprised 15 patients with no overlap to any model training data and constituted a patient-level held-out test set, used for primary performance evaluation. The remaining 59 patients had contributed archival tissue blocks to either the HE or OCT3/4 model training. The second evaluation set comprised all 74 patients of the second dataset and was used as a full-cohort evaluation to assess pipeline behavior on a broader sample. This cohort is not a patient-level held-out test set, but a slide-level split, providing a larger sample for assessing the consistency of pipeline performance across the entire study population.

### Reference standard

The reference standard is the Bergmann-Kliesch Score assessed by one of three board-certified uropathologists (JK, HR, PJW) for every slide during their routine clinical reporting over the retrospectively examined length of time. Every slide for every patient was scored and entered in the database of the SBB’s archive. The Bergmann-Kliesch Score is a histological scoring system used to quantify the state of spermatogenesis, representing the percentage of tubules with elongated spermatids divided by 10, yielding values between 0 and 10 [7]. Neither the AI pipeline nor the concept of the study existed during manual scoring, blinding the uropathologists to the AI predictions. The reference standard reflects real clinical circumstances.

### AI pipeline

All models were implemented and trained in Python (v3.8.10) using fastai (v2.7.18) [24] built on top of PyTorch (v1.13.1) [25]. Image registration was performed with SimpleITK (v2.5.3) [26] and scikit-image (v0.18.1) [27]. Bergmann-Kliesch Score calculation employed Shapely (v2.0.7) for spatial intersection computation [28]. WSIs were accessed via OpenSlide (v1.4.1) [29].

### Tubular segmentation (HE Model)

The tubular segmentation model was developed using a U-Net with a ResNet-34 backbone, which was pretrained on ImageNet. The model was implemented and trained using the fastai deep learning library [24]. In total, 5,633 seminiferous tubules were manually annotated by a doctoral researcher across the 60 HE-stained training sections (median 94 per slide, range 15–233), following structured training and quality review by an experienced uropathologist. The slides were split at the slide level into 48 sections for training and 12 sections for validation (80/20 split). Patches of 600 × 600 pixels were extracted at pyramid level 1 of the WSI pyramid (0.497 µm/pixel), yielding 3,135 training patches and 624 validation patches. Pixel-level segmentation was formulated as a three-class problem comprising background, tissue, and seminiferous tubule. Training augmentations included random flips, brightness, and contrast adjustments. The model was trained for 150 epochs with the cross-entropy loss being used as the optimization objective and the multi-class Dice coefficient (DiceMulti) as the evaluation metric. The best performing model based on lowest validation loss was retained for downstream inference and reached a multi-class Dice coefficient of 0.897 on the validation set. After segmentation, the resulting tubular masks were post-processed using morphological operations (minimum filter, opening, closing) and watershed-based separation to split adjacent tubules, followed by removal of connected components smaller than 6,000 µm^2^ to discard segmentation artefacts [30, 31].

### Spermatid detection (OCT3/4 Model)

The spermatid detection model utilized the same pretrained architecture as the tubule segmentation model. A total of 14,226 elongated spermatids were manually annotated across 11 OCT3/4 immunohistochemically stained training sections (median 736 per slide, range 37-4,352), following training and quality review by an experienced uropathologist (JK). The remaining four training sections were scored with a score of 0 and had therefore no elongated spermatids. Image patches of 224 × 224 pixels were extracted at native resolution (0.2486 µm/pixel), retaining only patches with at least 10% tissue coverage. Patches from the 11 spermatogenesis-positive slides were split 80/20 between training and validation. Patches from the four slides with a Bergmann-Kliesch Score of 0 were assigned at the slide level with two slides used for training and two for validation. This ensured a hybrid split that preserved patch-level diversity and slide-level evaluation of the negative class.

Pixel-level detection was formulated as a binary task comprising spermatid and non-spermatid tissue. Given the strong class imbalance due to the small size of the elongated spermatids, a weighted cross-entropy loss with a class weight of 26.9 for the spermatid class was used. This was calculated using the ratio of the number of pixels assigned to non-spermatid tissue and spermatid tissue. Augmentations included random flips, brightness, contrast, saturation and hue adjustments. The model was trained for 100 epochs and the model with the lowest validation loss was retained for downstream inference. The model achieved a soft Dice coefficient of 0.284 for the spermatid class on the validation set. While this metric reflects only an intermediate processing step, the downstream pipeline performance demonstrated strong agreement with pathologist-assigned scores. Spermatid detections were converted to polygonal contours and approximated using the Douglas-Peucker algorithm [32]. Contours with fewer than five vertices were discarded as detection artefacts.

### Image registration

**Fig. 2:**
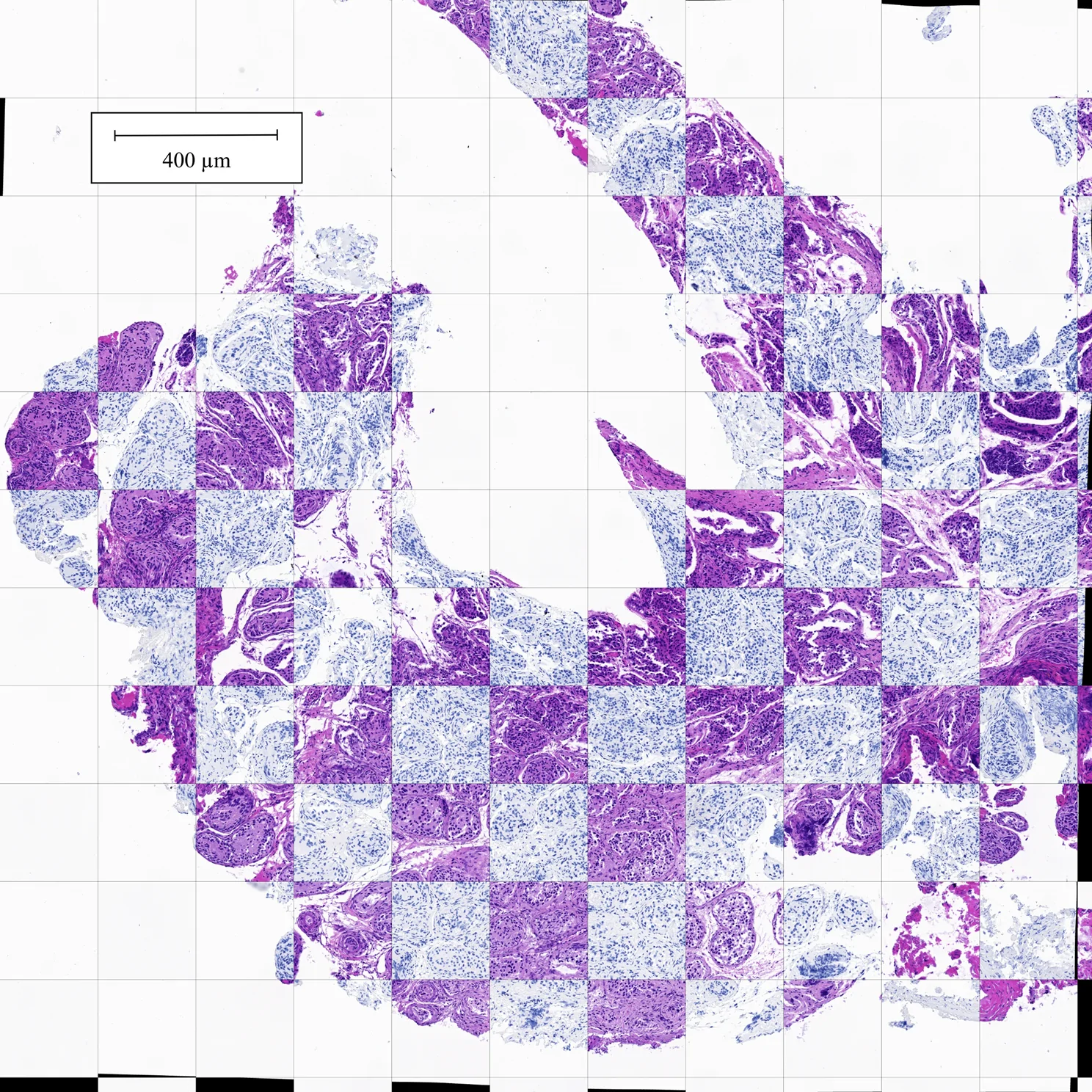
“Checkerboard visualization of multimodal image registration between paired HE and OCT3/4 sections.” Legend: “Checkerboard overlay of the registered haematoxylin-eosin (HE) and OCT3/4 immunohistochemically stained serial sections. Alternating tiles display the two modalities after image registration, allowing local alignment to be assessed across the tissue section. Continuous transitions of seminiferous tubule contours and tissue borders indicate accurate spatial correspondence in this representative case. This registration step is required to transfer OCT3/4-based spermatid detections into the coordinate space of the HE section and assign them to the corresponding segmented seminiferous tubules”

Spatial correspondence between paired HE and OCT3/4 sections was established by a two-stage multimodal image registration using SimpleITK [26]. A rigid alignment based on Sobel-gradient representations of both modalities was followed by a B-spline transform to account for residual non-linear tissue deformations between serial cuts [33]. Spermatid detections were subsequently propagated from OCT3/4 to HE coordinates by applying the combined transform. An example of the registration result is shown in Fig. 2.

### Bergmann-Kliesch Score calculation

**Fig. 3:**
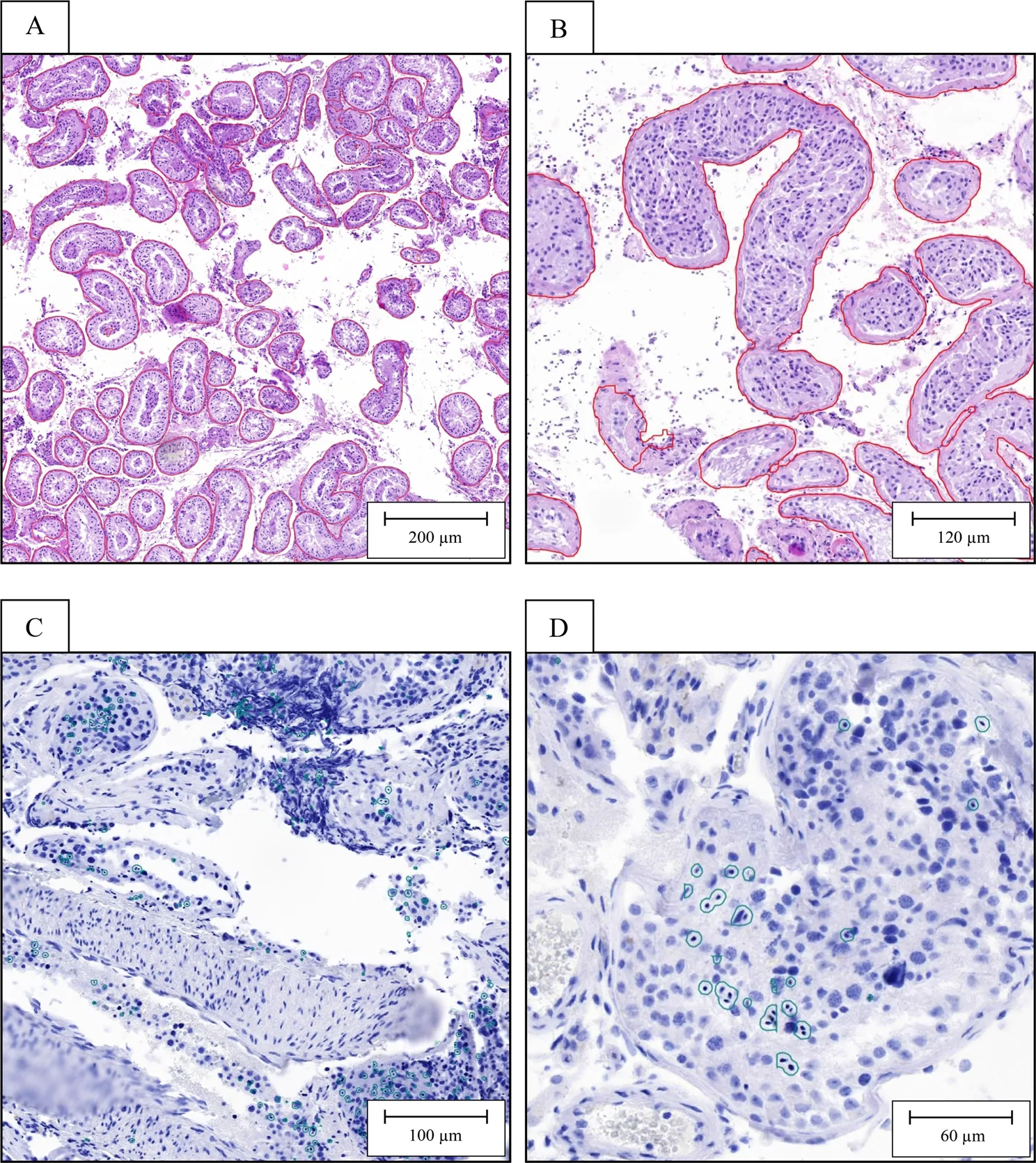
“Representative segmentation and detection examples on HE and OCT3/4 sections.” Legend: “Outputs of both segmentation models on WSI regions of the test cohort. (**A**) Overview of an HE-stained section with seminiferous tubules outlined by the tubular segmentation model. (**B**) Higher magnification HE example showing tubule segmentation in a region with closely adjacent and irregularly shaped tubules. Some neighboring tubules are not completely separated. Exact contour-level separation of every adjacent tubule is not required for downstream Bergmann-Kliesch Score calculation, since this relies on tubule-level assignment of spermatid detections. (**C**) Overview of an OCT3/4 immunohistochemically stained section, demonstrating that elongated spermatids are detected predominantly within seminiferous tubules. (**D**) Higher magnification OCT3/4 example illustrating detection of individual elongated spermatids and small clusters. Occasional false-positive detections may occur, including detections near surrounding elongated spermatids or morphologically similar structures. This limitation was addressed with the per-tubule threshold comparison for tubule classification”

After post-processing of the raw segmentation output and registration of the two modalities, the Bergmann-Kliesch Score was calculated automatically. Each spermatid detection was tested for spatial overlap with all tubular polygons using a spatial index implemented in Shapely [28]. The final Bergmann-Kliesch Score per slide was computed as the proportion of positive tubules among all detected tubules, multiplied by ten, yielding values between 0 and 10 in accordance with the original definition by Bergmann and Kliesch [7]. Examples of tubular segmentation are shown in Fig. 3A and B. Spermatid detections are shown in Fig. 3C and D.

### Statistical analysis

Agreement between AI-derived and pathologist-assigned Bergmann-Kliesch Scores was assessed separately on the patient-level held-out test set and the full evaluation cohort. The held-out test set was considered the primary performance evaluation, whereas analysis of the full cohort was considered supportive because patients from this cohort could have contributed archival tissue to model development. Continuous agreement was evaluated using Pearson correlation, Spearman rank correlation, Lin’s concordance correlation coefficient (CCC), mean absolute error (MAE), root mean squared error (RMSE), and mean bias. Bland-Altman analysis was used to assess agreement and calculate the 95% limits of agreement. Ninety-five percent confidence intervals were calculated for the main performance metrics. For Pearson correlation, confidence intervals were calculated using Fisher’s z transformation. For CCC, Spearman correlation, MAE, RMSE, and bias, bootstrap confidence intervals based on 10,000 resamples were used. Binary classification at the clinical cutoff of Bergmann-Kliesch Score ≥ 1 was evaluated using sensitivity, specificity, positive predictive value (PPV), negative predictive value (NPV), and accuracy with exact 95% confidence intervals.

A per-tubule spermatid threshold sensitivity analysis was performed post hoc across cutoff values of 1, 2, 3, 5, 7, and 10 detected spermatids per tubule. This was evaluated on both the full evaluation cohort (*n* = 74) and the patient-level held-out test set (*n* = 15). The threshold ultimately reported was identified from this empirical analysis rather than pre-specified prior to test-set evaluation. Statistical analyses were performed using scikit-learn (v0.24.1) [27], SciPy (v1.4.1) [34], and NumPy for bootstrap resampling. The study was reported in accordance with the Checklist for Artificial Intelligence in Medical Imaging (CLAIM) 2024 update [35] and the completed checklist is provided as Additional file 5.

### Runtime performance

Pipeline runtime was benchmarked on an NVIDIA GeForce RTX 3090 (24 GB VRAM). The total end-to-end processing time per WSI pair was approximately 25 minutes. Multimodal image registration (rigid alignment followed by B-spline deformable refinement via SimpleITK) was the most time-intensive step, requiring approximately 16 minutes per WSI pair. Reading whole-slide images from the proprietary scanner format, tissue masking, and patch or tile creation accounted for approximately 8 minutes. Segmentation inference for both the tubule and spermatid model combined required less than 1 minute. Reverse projection and Bergmann-Kliesch Score computation required approximately 1 minute.

## Results

The final evaluation cohort comprised 74 patients, including 15 patients in the patient-level held-out test set. In the full cohort, the median pathologist-assigned Bergmann-Kliesch Score was 1.00 (interquartile range (IQR) 0.00–8.00; range 0.00–10.00), with 35 cases below and 39 cases at or above the clinical cutoff of 1. In the held-out test set, the median reference score was 0.50 (IQR 0.00–7.50; range 0.00–10.00), with 8 cases below and 7 cases at or above the clinical cutoff.

**Fig. 4:**
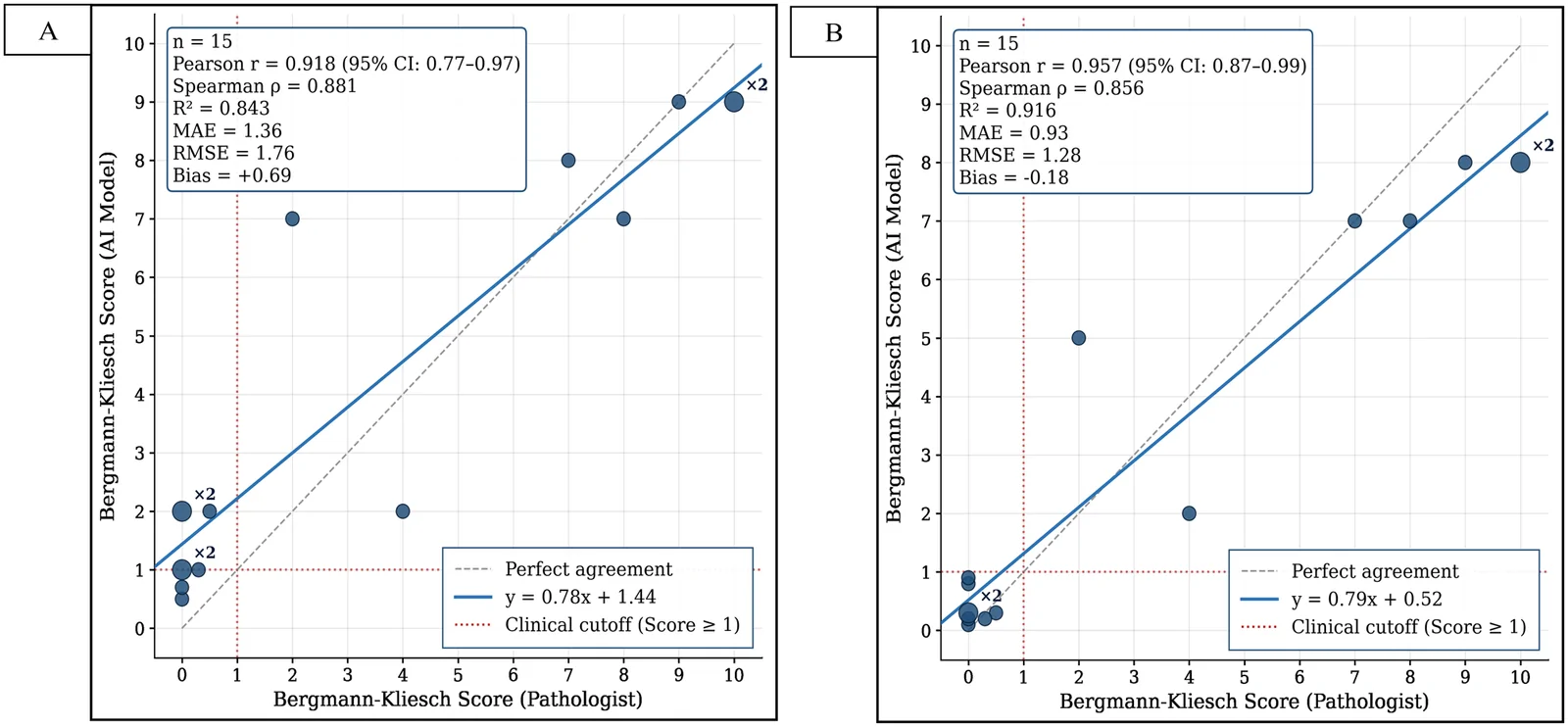
“Comparison of AI-derived vs. pathologist-assigned Bergmann-Kliesch scores on the test set.” Legend: “Scatter plots comparing AI-derived and pathologist-assigned Bergmann-Kliesch scores in the patient-level held-out test set (*n* = 15). Each point represents one patient. Point size reflects the number of overlapping cases at identical coordinates (xN annotation). The dashed grey line indicates perfect agreement, the blue line indicates the regression fit, and the red dotted lines indicate the institutional clinical cutoff for ICSI recommendation (Bergmann-Kliesch Score ≥ 1). (**A**) Results using a threshold of at least one detected elongated spermatid per seminiferous tubule to define a positive tubule. This configuration showed high correlation but produced a systematic positive bias. (**B**) Results using a threshold of at least two detected elongated spermatids per tubule. This threshold reduced bias and improved agreement with the pathologist-assigned reference scores. Statistical metrics are shown in each panel”

The AI-derived Bergmann-Kliesch Scores showed strong agreement with manually assigned reference scores on the patient-level held-out test set (*n* = 15), with the threshold of a positive tubule having equal to or above one elongated spermatid (Pearson *r* = 0.918; 95% CI: 0.77–0.97; Spearman ρ = 0.881; R^2^ = 0.843; MAE = 1.36; RMSE = 1.76). The correlation coefficient comparing reference Bergmann-Kliesch Scores with AI-derived scores is shown in Fig. 4A. However, this configuration produced a systematic positive bias (bias = 0.69) driven by overestimation in cases with absent spermatogenesis, due to sporadic false-positive detections. Increasing the threshold to ≥ 2 detected spermatids per seminiferous tubule substantially reduced this bias and improved overall agreement (Fig. 4B). On the held-out test set, Pearson correlation was 0.957 (95% CI: 0.873–0.986), Spearman correlation was 0.856 (95% CI: 0.517–0.967), and Lin’s CCC was 0.940 (95% CI: 0.852–0.977). The MAE was 0.93 (95% CI: 0.51–1.40), the RMSE was 1.28 (95% CI: 0.76–1.74), and the mean bias was −0.18 (95% CI: −0.79 to 0.47). Bland-Altman analysis showed 95% limits of agreement from −2.76 to 2.40, as shown in Additional file 4B.

**Fig. 5:**
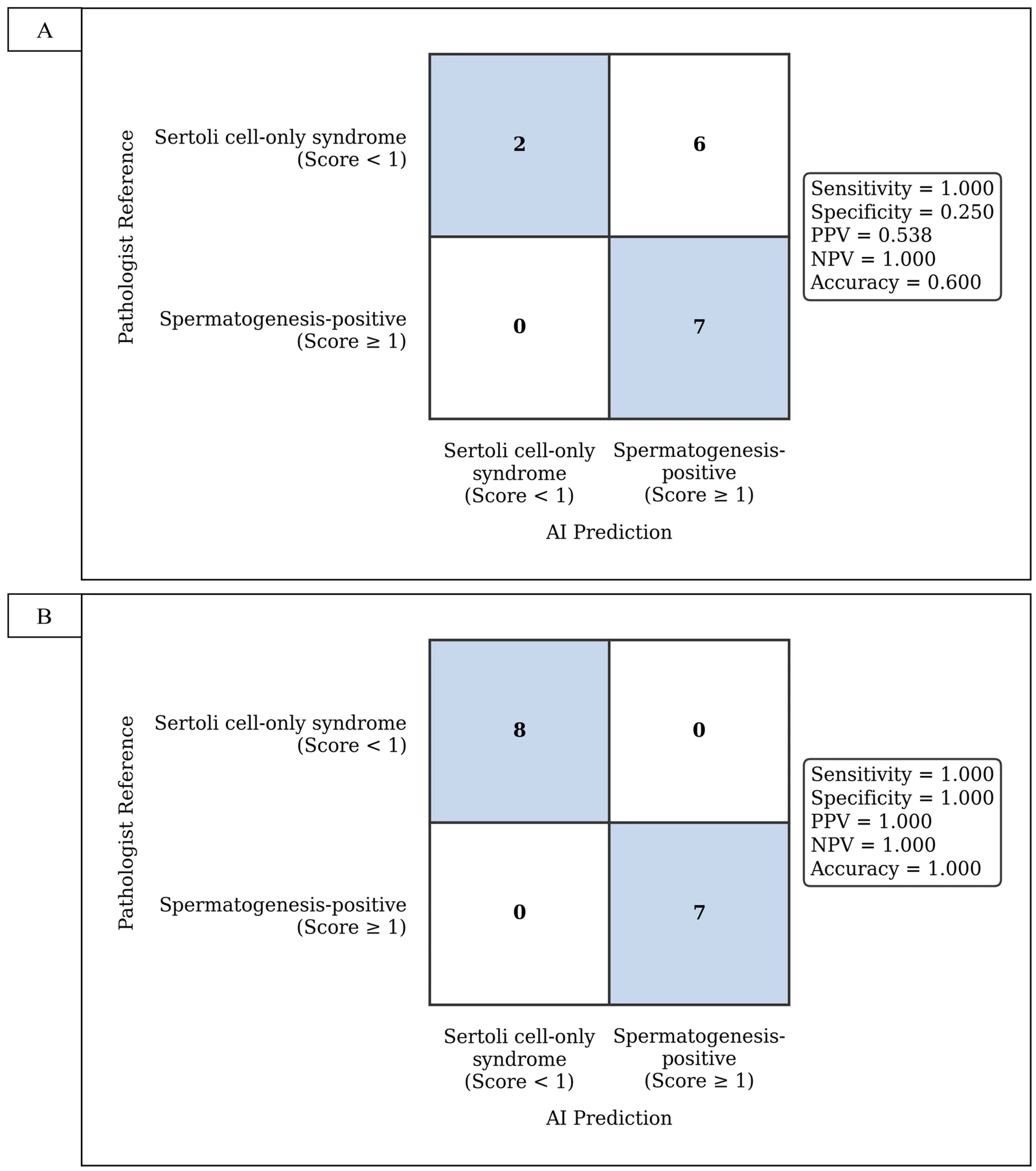
“Classification performance at the clinical cutoff Bergmann-Kliesch Score ≥ 1.” Legend: “Confusion matrices comparing AI-derived and pathologist-assigned classifications on the patient-level held-out test set (*n* = 15). Cases were classified at the institutional clinical cutoff of Bergmann-Kliesch Score ≥ 1, separating spermatogenesis-positive cases from Sertoli cell-only syndrome cases. (**A**) Using a threshold of at least one detected spermatid per tubule, the pipeline correctly identified all spermatogenesis-positive cases but produced six false-positive classifications among Sertoli cell-only syndrome cases. (**B**) Using a threshold of at least two detected spermatids per tubule, the pipeline showed complete agreement with the pathologist-assigned reference standard, with sensitivity, specificity, positive predictive value, negative predictive value, and accuracy each reaching 1.00”

Across the full evaluation cohort (*n* = 74), agreement at the adopted threshold remained strong. Pearson correlation was 0.851 (95% CI: 0.772–0.904), Spearman correlation was 0.829 (95% CI: 0.758–0.876), and Lin’s CCC was 0.820 (95% CI: 0.735–0.886). The MAE was 1.52 (95% CI: 1.17–1.89), the RMSE was 2.20 (95% CI: 1.76–2.60), and the mean bias was −0.21 (95% CI: −0.71 to 0.29). Bland-Altman analysis showed 95% limits of agreement from −4.52 to 4.10 (Additional file 4A). At the clinical cutoff of Bergmann-Kliesch Score ≥ 1, the pipeline achieved a sensitivity of 97.4% (95% CI: 86.5–99.9%), specificity of 62.9% (95% CI: 44.9–78.5%), positive predictive value of 74.5% (95% CI: 60.4–85.7%), negative predictive value of 95.7% (95% CI: 78.1–99.9%), and accuracy of 81.1% (95% CI: 70.3–89.3%). The sensitivity analysis across all tested per-tubule thresholds identified two spermatids per tubule as the highest-agreement configuration on this broader cohort (Additional file 2). It yielded the highest correlation and lowest mean absolute error among all tested values, whereas agreement progressively deteriorated at higher thresholds. Although seminiferous tubules are conventionally considered positive when containing at least one elongated spermatid, a threshold of ≥ 2 automatically detected elongated spermatids per tubule showed the highest concordance with the pathologists’ annotations and achieved perfect agreement in the patient-level held-out test set. The comparison in performance when applying the institutional clinical cutoff of Bergmann-Kliesch Score ≥ 1 is depicted in Fig. 5A and 5B. The AI pipeline using ≥ 2 detected spermatids per seminiferous tubule correctly classified all 15 cases in the held-out test set, identifying all 7 spermatogenesis-positive cases and all 8 SCOS cases (sensitivity = 1.00; specificity = 1.00; PPV = 1.00; NPV = 1.00).

The routine reference standard was assigned by one of three board-certified uropathologists (JK: *n* = 35; HR: *n* = 22; PJW: *n* = 17). To assess whether AI performance varied by reporting pathologist, we performed a pathologist-stratified analysis on the full evaluation cohort using the final AI score definition (a threshold of at least two detected spermatids per seminiferous tubule). At the clinical cutoff of Bergmann-Kliesch Score ≥ 1, concordance between AI-derived and pathologist-assigned classification was comparable across all three observers (JK: 29/35, 82.9%; HR: 17/22, 77.3%; PJW: 14/17, 82.4%; Additional file 3), with overlapping 95% Wilson confidence intervals. Discordant cases were almost exclusively false-positive AI classifications, with only a single false-negative case (in the PJW subgroup). Continuous agreement metrics showed a similar overall pattern across reporting pathologists. Mean absolute error was 1.54 for JK, 1.14 for HR, and 1.97 for PJW, while Pearson correlations were 0.856, 0.912, and 0.774, respectively. Mean bias was 0.00 for JK, −0.04 for HR, and −0.86 for PJW. Given the limited subgroup sizes, these analyses are considered descriptive and did not indicate a consistent reporting-pathologist-specific failure pattern.

## Discussion

We developed and validated an automated, multimodal AI-based pipeline for assessment of the Bergmann-Kliesch Score on whole-slide images from testicular biopsies. On a patient-level held-out test set of 15 patients, AI-derived scores showed strong correlation with pathologist-assigned reference scores. At the clinical decision cutoff used in our institution to recommend ICSI (Bergmann-Kliesch Score ≥ 1), the AI correctly classified all 15 cases. On the broader evaluation cohort of 74 patients, the agreement remained robust.

AI applications in andrology have so far primarily addressed other modalities or other steps of the patient pathway. Hu et al. predicted testicular histology non-invasively from grayscale ultrasound [16]. Wu et al. detected motile sperm in fresh TESE aspirates during embryology workup [17]. Ito et al. automated Johnsen scoring from HE-stained sections using a cloud-based classifier [18]. Automated per-tubule analysis of testicular WSIs has also been demonstrated in preclinical settings. This includes deep-learning-based spermatogenic staging in non-human primates [19] and cell- and tubule-level classification frameworks in rodents [20, 21]. Most recently, Kockwelp et al. introduced MARTHA, a deep-learning framework integrating passive eye-tracking with neural networks to generate large-scale annotated datasets of human testicular tubules and cell nuclei [22]. Building on this prior work, we present the first fully automated, WSI-based, per-tubule implementation of the Bergmann-Kliesch Score in human diagnostic biopsies, with pathologist-verifiable output. Our work extends this line of research from preclinical models to human clinical decision-making, and from generic tissue quantification to the direct automation of an established clinical scoring system that gates the ICSI recommendation. Our methodological approach utilizes image segmentation to obtain per-tubule predictions. These can then be verified by a pathologist directly on the slide. We combined two staining modalities. HE-staining for the segmentation of seminiferous tubules and OCT3/4 immunohistochemistry for the detection of elongated spermatids, through multimodal image registration.

The way OCT3/4 is used in our pipeline deserves particular attention. Although OCT3/4 immunohistochemistry is primarily used to detect germ cell neoplasia in situ (GCNIS) [23], we exploited its characteristic staining pattern for automated spermatid detection. When comparing OCT3/4-stained sections with their HE counterparts, we consistently observed that elongated spermatids stand out far more clearly in OCT3/4 than in HE at the same magnification (Additional file 1). We leveraged this property for automated detection. To our knowledge, this is the first report of using OCT3/4 immunohistochemistry (with target structures identified through negative staining) for automated spermatid detection. The approach is directly applicable in institutions such as ours where OCT3/4 immunohistochemistry is part of the routine workup for every testicular biopsy. In centers where this staining is not routinely performed, an additional staining step would be required before the pipeline could be applied.

A central methodological consideration in our pipeline was the definition of a positive tubule. The original Bergmann-Kliesch Score is defined qualitatively. A tubule is considered positive when elongated spermatids are visually identifiable within it, without a numerical detection threshold [7]. For an automated pixel-based pipeline, this criterion must be translated into a quantitative rule, since detector outputs may include false-positive predictions that an experienced pathologist would disregard.

The direct translation of the original criterion with at least one detected spermatid per tubule already produced a high correlation with the pathologist scores on the test set (Pearson *r* = 0.918, Fig. 4A). However, this configuration came with a systematic positive bias (bias = 0.69), caused by overestimation in cases with absent spermatogenesis. Under this threshold, six of the eight SCOS-cases received non-zero AI scores and were misclassified as spermatogenesis-positive at the clinical cutoff (Fig. 5A). In clinical practice, such false-positives would carry real consequences. Female partners of men in whom successful sperm retrieval is not realistically possible would still be referred to ICSI, undergoing the procedure without viable sperm being available. This would expose them to unnecessary procedural burden and false therapeutic hope.

A modest revision of the threshold, now requiring at least two intersecting spermatid detections per tubule, substantially reduced this bias without compromising the detection of spermatogenesis-positive cases (sensitivity = 1.00; Fig. 4B; Fig. 5B). The sensitivity analysis on the full evaluation cohort (*n* = 74) confirmed the same threshold as optimal, yielding the highest correlation and lowest mean absolute error across all tested values. Agreement deteriorated progressively at higher thresholds (Additional file 2). The consistency of this finding both on the small held-out test set and on the broader full evaluation cohort suggests that the chosen threshold reflects a property of the detection task itself, rather than an artefact of the small test set. However, as this threshold was identified from an empirical sensitivity analysis rather than pre-specified, prospective validation on independent external cohorts will be required before deployment in routine clinical workflows.

Our architectural choice was guided by the specific nature of the segmentation task rather than by current advancements in machine learning. For both the tubular segmentation and the spermatid detection model, we used a U-Net with a ResNet-34 backbone. This is a well-established combination in medical image analysis, especially for pixel-level segmentation tasks on histopathological WSIs [36, 37].

We deliberately did not adopt larger transformer-based architectures or pathology foundation models. Such models have shown strong performance across a wide range of histopathology tasks. This includes slide-level and patch-level applications as well as fine-tuning with task-specific heads, class weighting and dedicated validation [38, 39]. However, well-established CNN-based segmentation architectures such as U-Net have repeatedly achieved comparable or superior performance on narrowly defined, pixel-level histopathology tasks of limited data scale [40]. As our setting matched these conditions with a small training cohort and a highly localized detection target, we aimed for a transparent and computationally efficient pipeline suitable for routine clinical workflows. In this context, a U-Net with a pretrained ResNet-34 backbone offered an appropriate balance between model capacity and task specificity. The strong agreement with pathologist-assigned scores on the test set and the perfect classification at the clinical cutoff support this choice.

The comparatively low pixel-level Dice coefficient of the spermatid detection model requires careful interpretation. At first glance, a soft Dice coefficient of 0.284 appears modest for a segmentation model. However, elongated spermatids are extremely small structures and occupy only a minimal fraction of each image patch. Because of this, even small differences between manual annotation and model prediction can strongly reduce the Dice score, even if the prediction is still close enough to be useful for the downstream analysis.

This is relevant because the aim of the pipeline is not to draw a perfect outline around every single spermatid. For the Bergmann-Kliesch Score, the key question is whether a seminiferous tubule contains elongated spermatids. Therefore, the pipeline summarizes spermatid detections at the level of individual tubules after image registration. The final score is consequently less dependent on perfect pixel-level overlap and more dependent on assigning detections to the correct tubules.

The strong agreement between AI-derived and pathologist-assigned scores supports this interpretation. Although the spermatid model had a modest Dice score, the complete pipeline showed robust agreement with the reference standard and correctly classified all cases in the held-out test set at the clinical cutoff after applying the two-spermatid threshold. This suggests that, for this clinical use case, tubule-level performance is more relevant than the isolated pixel-wise segmentation performance of the spermatid model.

The present study has several limitations. First, this was a retrospective, monocentric study, and all slides were obtained from a single institution using local tissue processing, staining and scanning. Although this provides a controlled setting for model development, it limits the generalizability of the results to other centers with different practices.

Second, the patient-level held-out test set was relatively small, comprising 15 patients. This test set is important because these patients had no overlap with the model development data. However, the small sample size limits the precision of the performance estimates. This is especially true for the binary classification at the clinical cutoff. The perfect classification performance in this subset should therefore be interpreted cautiously and requires confirmation in larger external cohorts.

Third, the reference standard was based on routine clinical Bergmann-Kliesch Scores assigned during diagnostic reporting. This reflects real-world practice but is not the same as a prospective standard generated by multiple pathologists specifically for this study. Manual testicular biopsy assessment is known to show interobserver variability [10]. Therefore, part of the disagreement between AI-derived and reference scores may reflect variability in the reference standard itself rather than errors of the pipeline alone.

Fourth, the current end-to-end processing time of approximately 25 minutes per WSI pair is dominated by the image registration step. Segmentation inference for both models combined required less than 1 minute, suggesting that the computational bottleneck lies in the registration rather than in the deep learning components and could be addressed by more efficient registration frameworks.

Fifth, the pipeline depends on accurate registration between serial HE and OCT3/4 sections. The sections were 2 µm thick and immediately consecutive, and the image registration combined rigid alignment with B-spline warping, but local tissue deformations, artefacts, and tissue loss may still lead to imperfect spatial correspondence. Such errors could cause spermatid detections to be assigned to the wrong tubule or to fall outside the corresponding tubule. Registration quality remains a critical technical step in this pipeline.

Finally, the present study evaluated agreement with pathologist-assigned Bergmann-Kliesch Scores but did not assess direct clinical outcome measures. We did not analyze whether AI-derived Bergmann-Kliesch Scores predict sperm retrieval rates, ICSI success, pregnancy, or live birth outcomes. Therefore, the current results show that the pipeline can reproduce the histopathological score, but they do not yet prove that the AI-derived score improves clinical decision-making or patient outcomes.

Future work should focus on external validation in larger multicentric cohorts. Slides from different laboratories with different WSI processing protocols should be evaluated to test the robustness and generalizability of the pipeline. Standardized reference scoring, ideally based on consensus assessment by several experienced pathologists, should be used.

Additionally, future studies should investigate the relationship between AI-derived Bergmann-Kliesch Scores and clinical outcome parameters. These parameters should include sperm retrieval rates, ICSI outcomes and pregnancy rates. This would clarify whether Bergmann-Kliesch Score assessment can both reproduce routine histopathological scoring and improve clinical decision-making in men with NOA.

Further technical development would be the integration of quality control steps for segmentation and registration. Detection and flagging of failed registration, tissue artefacts or low-confidence predictions could improve safety before clinical implementation. In addition, future versions of the pipeline could explore whether the spermatid detection step can be transferred to other staining protocols or eventually simplified toward a single-slide workflow.

Our findings suggest that automated Bergmann-Kliesch Score assessment from digitized testicular biopsies is technically feasible and can reproduce routine pathologist scoring with high agreement. The method should currently be understood as a decision-support tool rather than a replacement for expert assessment. Its main potential lies in improving reproducibility, reducing manual workload, and providing transparent tubule-level outputs that can be validated individually by a pathologist.

## Conclusions

This study presents a proof-of-concept AI-based pipeline for Bergmann-Kliesch Score assessment from paired HE and OCT3/4-stained whole-slide images of testicular biopsies. The pipeline showed strong agreement with pathologist-assigned reference scores and correctly classified all cases in the held-out test set at the clinically relevant cutoff after applying a two-spermatid-per-tubule threshold. These findings support the feasibility of automated, tubule-level Bergmann-Kliesch Score assessment as a reproducible decision-support tool in TESE workflows.

## Electronic supplementary material

Below is the link to the electronic supplementary material.

Supplementary Material 1: Enhanced visual contrast for spermatids in OCT3/4 section. Comparison of elongated spermatid visibility in paired haematoxylin-eosin (HE) and OCT3/4-stained serial sections. The same seminiferous tubule region is shown in both stains. Arrows indicate representative elongated spermatids that are more clearly discernible in the immunohistochemically stained section than in the corresponding HE section. The visual contrast was used to support manual annotation and automated detection of elongated spermatids in the OCT3/4 model.

Supplementary Material 2: Threshold sweep across the full evaluation cohort. Threshold sweep for defining a spermatid-positive seminiferous tubule in the full evaluation cohort (n = 74). Scatter plots compare pathologist-assigned and AI-derived Bergmann-Kliesch Scores across six tubular spermatid-count thresholds: (A) ≥1, (B) ≥2, (C) ≥3, (D) ≥5, (E) ≥7, and (F) ≥10 detected spermatids per tubule. Blue circles denote non-test patients with archival tissue overlap with the model development data (n = 59), and orange circles denote patients from the patient-level held-out test set (n = 15). Marker size and xN labels indicate overlapping observations at identical coordinates. The grey dashed line represents perfect agreement, the blue solid line shows the ordinary least-squares regression with its equation, and the red dotted lines indicate the clinical cutoff at Bergmann-Kliesch Score ≥1. Per-panel statistics include Pearson correlation (r) with 95% confidence interval, Spearman rank correlation (ρ), coefficient of determination (R2), mean absolute error (MAE), root mean square error (RMSE), and mean bias.

Supplementary Material 3: Pathologist-stratified classification at the clinical cutoff. Pathologist-stratified classification at the clinical cutoff of Bergmann-Kliesch Score ≥1 on the full evaluation cohort (n = 74), using the final AI score definition (threshold of at least two detected spermatids per seminiferous tubule). Stacked bars show the distribution of true positive, true negative, false positive, and false negative classifications per reporting pathologist, expressed as percentage of the pathologist’s cases (JK: Jens Kρllermann, n = 35; HR: Henning Reis, n = 22; PJW: Peter J. Wild, n = 17). Absolute counts are annotated within each stack segment. Diamond markers on the bars indicate the overall concordance (TP + TN) with 95% Wilson confidence intervals, illustrating overlapping confidence intervals across all three observers.

Supplementary Material 4: Bland-Altman analysis of AI-derived vs. pathologist-assigned Bergmann-Kliesch Scores. Difference (AI−Reference) plotted against the mean of both scores for (A) the full evaluation cohort (n = 74) and (B) the patient-level held-out test set (n = 15). The solid blue line indicates the mean bias, the dashed red lines indicate the 95% limits of agreement (LoA; bias±1.96 SD), and shaded bands represent 95% bootstrap confidence intervals for bias and LoA. On the full cohort, bias was −0.21 (95% CI: −0.71 to 0.29) with LoA from −4.52 to 4.10. On the test set, bias was −0.18 (95% CI: −0.79 to 0.47) with narrower LoA from −2.76 to 2.40.

Supplementary Material 5: CLAIM 2024 Checklist. Completed Checklist for Artificial Intelligence in Medical Imaging (CLAIM) 2024 update for the present study, indicating for each of the 44 CLAIM items whether it is addressed in the manuscript, the corresponding section, page, and a brief note describing where or how the item is addressed.

## Data Availability

Inference workflow, configuration template, and software requirements are available at [https://github.com/SGassenAI/bergmann-kliesch-ai-pipeline]. Trained model weights are available on reasonable request, subject to institutional and data protection requirements.

## Abbreviations

AI: Artificial intelligence
CCC: Concordance correlation coefficient (Lin’s)
CI: Confidence interval
CLAIM: Checklist for Artificial Intelligence in Medical Imaging
FFPE: Formalin-fixed, paraffin-embedded
GCNIS: Germ cell neoplasia in situ
HE: Haematoxylin-eosin
ICSI: Intracytoplasmic sperm injection
IQR: Interquartile range
LoA: Limits of agreement
LLM: Large language model
MAE: Mean absolute error
NOA: Non-obstructive azoospermia
NPV: Negative predictive value
OCT3/4: Octamer-binding transcription factor 3/4
PPV: Positive predictive value
R^2^: Coefficient of determination
RMSE: Root mean squared error
SBB: Senckenberg BioBank
SCOS: Sertoli cell-only syndrome
SD: Standard deviation
TESE: Testicular sperm extraction
WSI: Whole-slide image
