# Supplementary material for: Automated Bergmann-Kliesch Score assessment in testicular biopsies using artificial intelligence-based whole-slide image analysis: a retrospective monocentric development and validation study
Source: Basic Clin Androl. 2026 Oct 6;36:27. doi: 10.1186/s12610-026-00329-x (PMC13640392; doi:10.1186/s12610-026-00329-x)
Supplement: Supplementary file 2 [file 12610_2026_329_MOESM2_ESM.pdf]

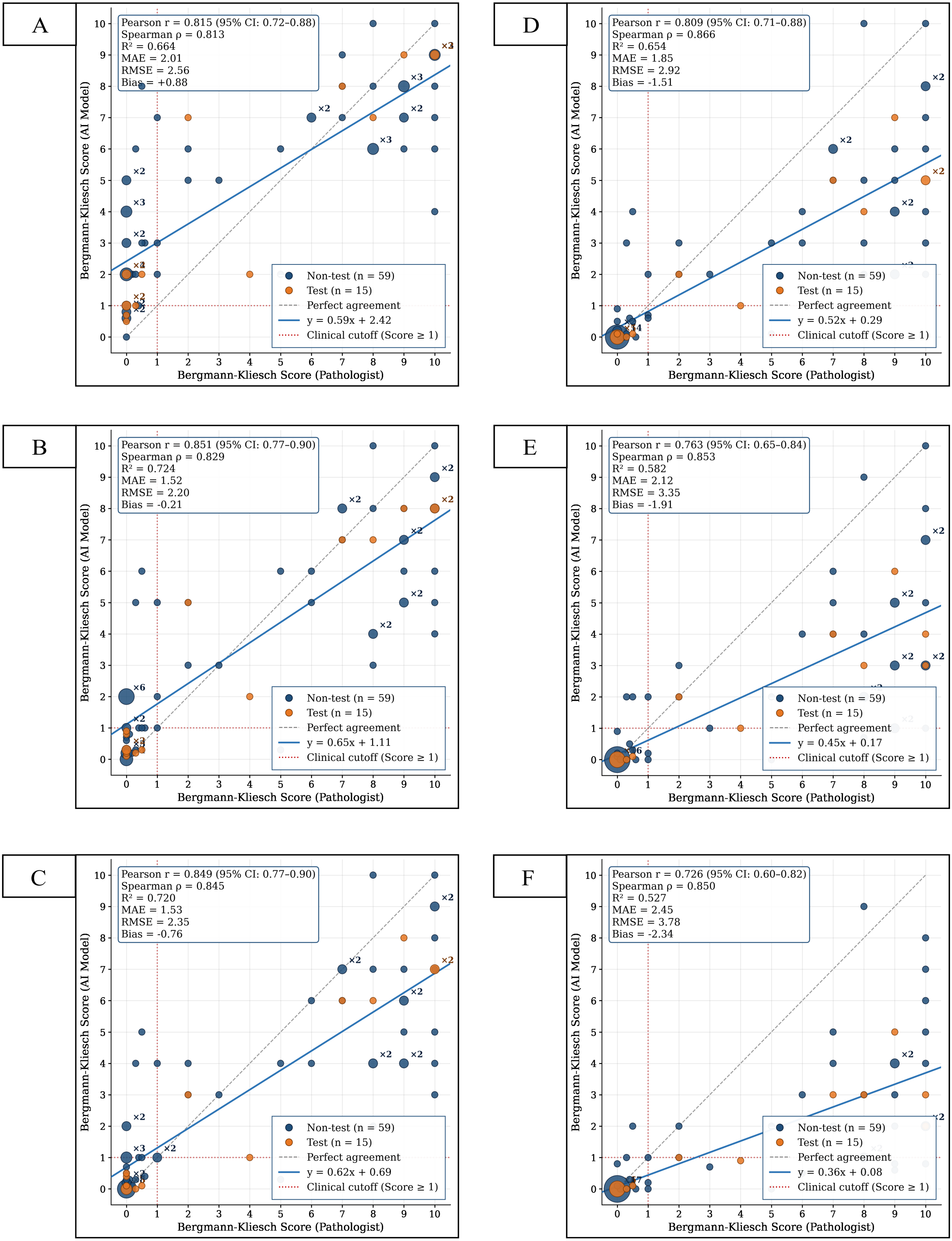
A
Pearson r = 0.815 (95% CI: 0.72–0.88)
Spearman ρ = 0.813
R² = 0.664
MAE = 2.01
RMSE = 2.56
Bias = +0.88
Non-test (n = 59)
Test (n = 15)
Perfect agreement
y = 0.59x + 2.42
Clinical cutoff (Score ≥ 1)
Bergmann-Kliesch Score (AI Model)
Bergmann-Kliesch Score (Pathologist)
B
Pearson r = 0.851 (95% CI: 0.77–0.90)
Spearman ρ = 0.829
R² = 0.724
MAE = 1.52
RMSE = 2.20
Bias = -0.21
Non-test (n = 59)
Test (n = 15)
Perfect agreement
y = 0.65x + 1.11
Clinical cutoff (Score ≥ 1)
Bergmann-Kliesch Score (AI Model)
Bergmann-Kliesch Score (Pathologist)
C
Pearson r = 0.849 (95% CI: 0.77–0.90)
Spearman ρ = 0.845
R² = 0.720
MAE = 1.53
RMSE = 2.35
Bias = -0.76
Non-test (n = 59)
Test (n = 15)
Perfect agreement
y = 0.62x + 0.69
Clinical cutoff (Score ≥ 1)
Bergmann-Kliesch Score (AI Model)
Bergmann-Kliesch Score (Pathologist)
D
Pearson r = 0.809 (95% CI: 0.71–0.88)
Spearman ρ = 0.866
R² = 0.654
MAE = 1.85
RMSE = 2.92
Bias = -1.51
Non-test (n = 59)
Test (n = 15)
Perfect agreement
y = 0.52x + 0.29
Clinical cutoff (Score ≥ 1)
Bergmann-Kliesch Score (AI Model)
Bergmann-Kliesch Score (Pathologist)
E
Pearson r = 0.763 (95% CI: 0.65–0.84)
Spearman ρ = 0.853
R² = 0.582
MAE = 2.12
RMSE = 3.35
Bias = -1.91
Non-test (n = 59)
Test (n = 15)
Perfect agreement
y = 0.45x + 0.17
Clinical cutoff (Score ≥ 1)
Bergmann-Kliesch Score (AI Model)
Bergmann-Kliesch Score (Pathologist)
F
Pearson r = 0.726 (95% CI: 0.60–0.82)
Spearman ρ = 0.850
R² = 0.527
MAE = 2.45
RMSE = 3.78
Bias = -2.34
Non-test (n = 59)
Test (n = 15)
Perfect agreement
y = 0.36x + 0.08
Clinical cutoff (Score ≥ 1)
Bergmann-Kliesch Score (AI Model)
Bergmann-Kliesch Score (Pathologist)
