# Supplementary material for: Automated Bergmann-Kliesch Score assessment in testicular biopsies using artificial intelligence-based whole-slide image analysis: a retrospective monocentric development and validation study
Source: Basic Clin Androl. 2026 Oct 6;36:27. doi: 10.1186/s12610-026-00329-x (PMC13640392; doi:10.1186/s12610-026-00329-x)

Pathologist-stratified classification at the clinical cutoff

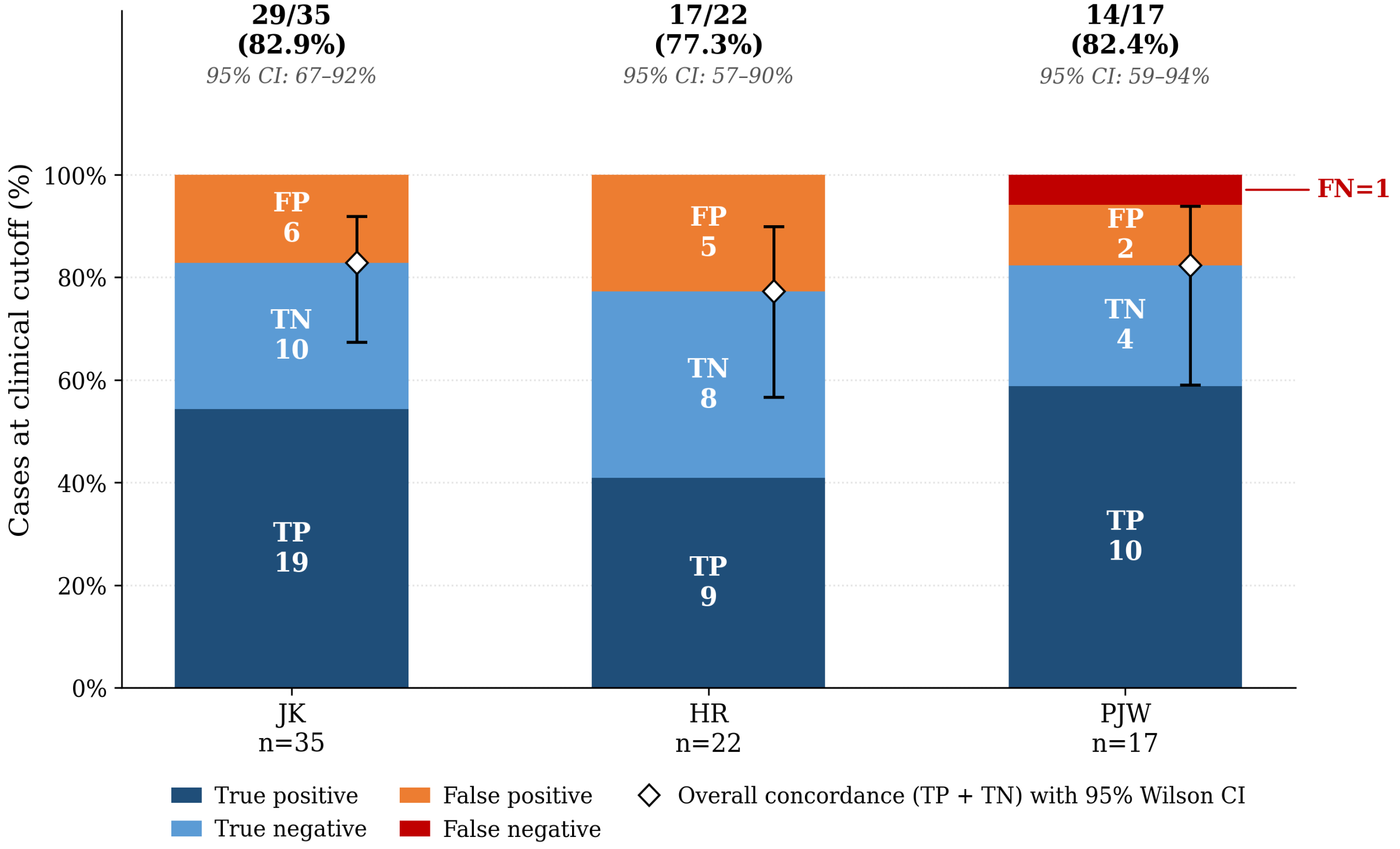

Supplement: Supplementary file 3 [file 12610_2026_329_MOESM3_ESM.pdf]
