# Supplementary material for: Automated Bergmann-Kliesch Score assessment in testicular biopsies using artificial intelligence-based whole-slide image analysis: a retrospective monocentric development and validation study
Source: Basic Clin Androl. 2026 Oct 6;36:27. doi: 10.1186/s12610-026-00329-x (PMC13640392; doi:10.1186/s12610-026-00329-x)
Supplement: Supplementary file 4 [file 12610_2026_329_MOESM4_ESM.pdf]

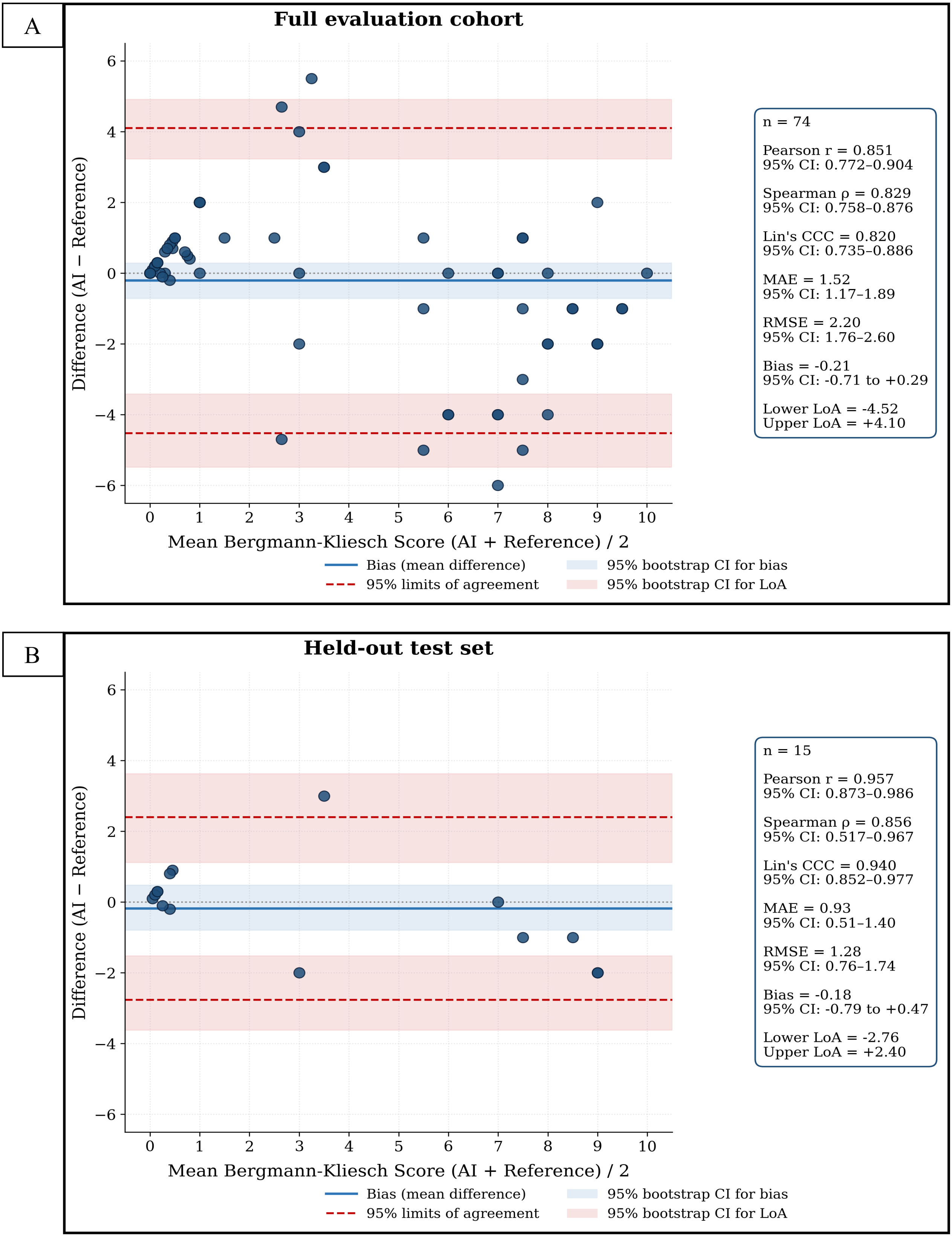
A
Full evaluation cohort
Difference (AI − Reference)
Mean Bergmann-Kliesch Score (AI + Reference) / 2
n = 74
Pearson r = 0.851
95% CI: 0.772–0.904
Spearman ρ = 0.829
95% CI: 0.758–0.876
Lin's CCC = 0.820
95% CI: 0.735–0.886
MAE = 1.52
95% CI: 1.17–1.89
RMSE = 2.20
95% CI: 1.76–2.60
Bias = -0.21
95% CI: -0.71 to +0.29
Lower LoA = -4.52
Upper LoA = +4.10
Bias (mean difference)
95% limits of agreement
95% bootstrap CI for bias
95% bootstrap CI for LoA
B
Held-out test set
Difference (AI − Reference)
Mean Bergmann-Kliesch Score (AI + Reference) / 2
n = 15
Pearson r = 0.957
95% CI: 0.873–0.986
Spearman ρ = 0.856
95% CI: 0.517–0.967
Lin's CCC = 0.940
95% CI: 0.852–0.977
MAE = 0.93
95% CI: 0.51–1.40
RMSE = 1.28
95% CI: 0.76–1.74
Bias = -0.18
95% CI: -0.79 to +0.47
Lower LoA = -2.76
Upper LoA = +2.40
Bias (mean difference)
95% limits of agreement
95% bootstrap CI for bias
95% bootstrap CI for LoA
