# Supplementary material for: Automated Bergmann-Kliesch Score assessment in testicular biopsies using artificial intelligence-based whole-slide image analysis: a retrospective monocentric development and validation study
Source: Basic Clin Androl. 2026 Oct 6;36:27. doi: 10.1186/s12610-026-00329-x (PMC13640392; doi:10.1186/s12610-026-00329-x)
Supplement: Supplementary file 5 [file 12610_2026_329_MOESM5_ESM.pdf]

Additional file 5

CLAIM 2024 Checklist

Checklist for Artificial Intelligence in Medical Imaging (CLAIM)

Title of study: Automated Bergmann-Kliesch Score Assessment in Testicular Biopsies Using Artificial Intelligence-Based Whole-Slide Image Analysis: A Retrospective Monocentric Development and Validation Study

Journal: Basic and Clinical Andrology

Corresponding Author: Prof. Dr. med. Peter J. Wild

Checklist Version: CLAIM 2024 Update (Tejani et al., Radiology: AI 2024; DOI: 10.1148/ryai.240300)

#	Item	Yes / No / NA	Page	Notes
**Title/Abstract**				
1	Identification as AI methodology study (title / abstract)	Yes	Title (page 1), Abstract (page 2-3)	“Artificial Intelligence-Based” in Title; “AI-based pipeline” and “U-Net models” in abstract
**Abstract**				
2	Summary of study design, methods, results, conclusions	Yes	Abstract (page 2-3)	Structured abstract; patient – level split explicit; data / code availability addressed in Data Availability statement
**Introduction**				
3	Scientific / clinical background, incl. intended use and role of AI approach	Yes	Introduction (page 5-6)	Clinical context of NOA and TESE workflow established; current practice explicitly mentioned (current scoring by uropathologist); prior AI work in andrology and preclinical testicular histology reviewed; intended role of AI as decision support for TESE / ICSI recommendation stated
4	Study aims, objectives and hypotheses	Yes	Introduction (page 6)	Study aims stated; development of an AI pipeline for automated Score assessment; not a hypothesis-driven prediction study but a data-driven methodological development, which is acceptable by CLAIM 2024
**Methods**				
Study Design				
5	Prospective or retrospective study	Yes	Title (page 1), Methods (page 7)	Retrospective study stated in Patient Cohort
6	Study goal	Yes	Introduction (page 6), Conclusions (page 23-24)	“Proof-of-concept AI-based pipeline for Bergmann-Kliesch Score assessment”
Data				
7	Data sources	Yes	Methods (page 7-10)	“Dataset derived from the archive of the SBB was used exclusively for model development”
8	Inclusion and exclusion criteria	Yes	Methods (page 7)	Inclusion and exclusion criteria stated in the Study Design and Patient Cohort section
9	Data preprocessing	Yes	Methods (page 10-13)	AI Pipeline subsection (software libraries with versions); Tubular Segmentation and Spermatid Detection subsections
10	Selection of data subsets	Yes	Methods (page 10-12)	Tubular Segmentation and Spermatid Detection subsections: Manual annotation with expert supervision described

11	De-identification methods	Yes	Declarations (page 25)	Ethics approval with approval by Ethics Committee
12	How missing data were handled	NA	-	Not applicable. Image-based analysis with complete WSI data for all 74 included patients; All exclusions (10 patients) documented at the level of inclusion criteria; not through data imputation
13	Image acquisition protocol	Yes	Methods (page 8)	Dataset and Tissue Processing subsection: Image acquisition protocolled with scanner, magnification, pixel resolution, section thickness, staining protocol all mentioned
Reference Standard				
14	Definition of methods used to obtain reference standard	Yes	Methods (page 10)	Reference Standard subsection: "Bergmann-Kliesch Score assessed by one of three board-certified uropathologists (JK, HR, PJW) for every slide during their routine clinical reporting"; Score definition with reference
15	Rationale for using the reference standard	Yes	Methods (page 10)	Reference Standard subsection: routine clinical scoring by board-certified uropathologists reflects real-world clinical circumstances
16	Source of reference standard annotations	Yes	Methods (page 10)	Reference standard subsection: "Every slide for every patient was scored and entered in the database of the SBB's archive" by board certified uropathologists
17	Annotation of test set	Yes	Methods (page 10)	Same reference standard applied to test set as to full cohort; uropathologists blinded to AI predictions
18	Measures of inter- and intrarater variability of features described by the annotators	No	Methods (page 10-12), Results (page 18), Additional files (page 33-34)	Manual training annotations performed by a single trained doctoral researcher under structured training and quality review by an experienced uropathologist; no formal interrater analysis on annotations was conducted; Reference-standard reproducibility across the three reporting uropathologists is addressed by a pathologist-stratified concordance analysis (**Additional file 3**)
Data Partitions				
19	How data were assigned to partitions	Yes	Methods (page 10-12)	Tubular segmentation section and Spermatid Detection section
20	Level at which partitions are disjoint	Yes	Methods (page 9-12)	Tubular model: slide-level split; Spermatid model: hybrid patch-level (positive slides) and slide-level (negative slides) split. Test cohort: "patient-level held-out test set" explicitly defined in Datasets subsection
Testing Data				

21	Intended sample size	No	-	Not formally calculated via power analysis. Sample size determined by all available cases in institutional archive between January 2019 and October 2024. Limitation is acknowledged in the Discussion (single-center retrospective study)
Model				
22	Detailed description of model	Yes	Methods (page 10-12)	Tubular Segmentation and Spermatid Detection subsections: U-Net architecture with ResNet-34 backbone pretrained on ImageNet; processing and post-processing all described
23	Software libraries, frameworks and packages	Yes	Methods (page 10)	AI Pipeline subsection
24	Initialization of model parameters	Yes	Methods (page 10-12)	Tubular Segmentation and Spermatid Detection subsections: ResNet-34 backbone pretrained on ImageNet; transfer learning explicitly stated
Training				
25	Details of training approach	Yes	Methods (page 10-12)	Tubular Segmentation and Spermatid Detection subsections: training, class weights, augmentations specified
26	Method of selecting the final model	Yes	Methods (page 10-12)	Both subsections: "The best performing model based on lowest validation loss was retained for downstream inference"
27	Ensembling techniques	NA	-	Not applicable. Single-model pipeline for each modality; no ensemble models used
Evaluation				
28	Metrics of model performance	Yes	Methods (page 14), Results (page 15-18)	Statistical analysis subsection; **Figures 4 and 5**
29	Statistical measures of significance and uncertainty	Yes	Methods (page 14), Results (page 15-18)	Statistical Analysis subsection specifies Fisher's z for Pearson correlation, 10,000 replicate bootstrap for CCC, Spearman, MAE, RMSE and bias, and exact 95% confidence intervals for classification metrics
30	Robustness or sensitivity analysis	Yes	Methods (page 14), Results (page 17)	Statistical Analysis subsection; per-tubule spermatid threshold sensitivity analysis performed across cutoff values 1, 2, 3, 5, 7, 10; results reported in Results section and visualized in **Additional file 2**
31	Methods for explainability or interpretability	Yes	Methods (page 10-13), Discussion (page 18-21)	Pipeline is interpretable by design: per-tubule predictions can be visually verified by pathologist directly on the slide (**Figure 3**); segmentation-based approach yields pathologist-verifiable output as emphasized in Discussion
32	Evaluation on internal data	Yes	Methods (page 9-10), Results (page 15-18)	Patient-level held-out test set (n = 15) with no overlap to training data; full evaluation cohort (n = 74) as secondary evaluation on broader sample. Both defined explicitly in Datasets subsection and reported in Results
33	Testing on external data	No	Discussion (page 22-23)	No external testing performed. Single-center design acknowledged as limitation in the Discussion;

				prospective external validation identified as necessary next step before clinical deployment
34	Clinical trial registration	NA	-	Not applicable. Study is a retrospective methodological development and validation study, not a prospective clinical trial
**Results**				
Data				
35	Numbers of patients or examinations included and excluded	Yes	Methods (page 7), Results (page 15)	Study Design and Patient Cohort subsections
36	Demographic and clinical characteristics of cases in each partition	Yes	Results (page 15)	Basic cohort characteristics given; broader clinical variables were not systematically extracted from routine records
Model Performance				
37	Performance metrics and measures of statistical uncertainty	Yes	Results (page 15-18)	Test set (n = 15): Pearson r = 0.957, MAE = 0.93, RMSE =1.28, bias = -0.18; perfect classification at cutoff; Full cohort (n = 74): Pearson r = 0.851, MAE = 1.52. Metrics benchmarked against reference standard (pathologist scores)
38	Estimates of diagnostic performance and their precision	Yes	Results (page 16-17)	Sensitivity, specificity, PPV, NPV, and accuracy with exact 95% confidence intervals reported at the clinical cutoff of Bergmann-Kliesch Score ≥ 1 for both the held-out test set and full evaluation cohort
39	Failure analysis of incorrect results	Yes	Results (page 15-18), Discussion (page 19-20)	Confusion matrix at clinical cutoff presented in **Figure 5**. Discussion analyzes systematic positive bias and threshold selection; **Additional file 2**
**Discussion**				
40	Study limitations	Yes	Discussion (page 20-23)	Study design, sample size, no external validation, post hoc threshold identification, technical limitations of image registration
41	Implications for practice, including intended use and/or clinical role	Yes	Discussion (page 18-23), Conclusions (page 23-24)	Discussion positions pipeline as decision-support tool for TESE/ICSI recommendation
**Other Information**				
42	Reference to full study protocol or additional technical details	Yes	Methods (page 10-14)	Detailed methodology fully documented in Methods section; **Additional file 1** and **Additional file 2** provide supplementary detail; CLAIM 2024 checklist provided as **Additional file 5**
43	Statement about availability of software, trained model and/or data	Yes	Declarations (page 25)	Data Availability subsection
44	Sources of funding and other support; role of funders	Yes	Declarations (page 25)	Declarations section includes funding statement and disclosure of any support
